# Three-Dimensional X-ray Imaging of β-Galactosidase Reporter Activity by Micro-CT: Implication for Quantitative Analysis of Gene Expression

**DOI:** 10.3390/brainsci11060746

**Published:** 2021-06-04

**Authors:** Olga Ermakova, Tiziana Orsini, Paolo Fruscoloni, Francesco Chiani, Alessia Gambadoro, Sabrina Putti, Maurizio Cirilli, Alessio Mezzi, Saulius Kaciulis, Miriam Pasquini, Marcello Raspa, Ferdinando Scavizzi, Glauco P. Tocchini-Valentini

**Affiliations:** 1 Adriano Buzzati-Traverso Campus, European Mouse Mutant Archive (EMMA), INFRAFRONTIER, Monterotondo Mouse Clinic (MMC), Italian National Research Council (CNR), Via Ramarini, 32, Monterotondo, 00015 Rome, Italy; tiziana.orsini@cnr.it (T.O.); paolo.fruscoloni@cnr.it (P.F.); francesco.chiani@cnr.it (F.C.); alessia.gambadoro@cnr.it (A.G.); sabrina.putti@cnr.it (S.P.); miriam.pasquini@emma.cnr.it (M.P.); marcello.raspa@cnr.it (M.R.); ferdinando.scavizzi@emma.cnr.it (F.S.); glaucotocchini@gmail.com (G.P.T.-V.); 2 Adriano Buzzati-Traverso Campus, Institute of Biochemistry and Cell Biology (IBBC), Italian National Research Council (CNR), Via Ramarini, 32, Monterotondo Scalo, 00015 Rome, Italy; maurizio.cirilli@cnr.it; 3 Institute for the Study of Nanostructured Materials, ISMN–CNR, Monterotondo Staz., 00015 Rome, Italy; alessio.mezzi@cnr.it (A.M.); saulius.kaciulis@ismn.cnr.it (S.K.)

**Keywords:** mouse brain, gene expression, *lacZ* reporter, X-ray imaging, pontocerebellar hypoplesia, Tsen54, mouse phenotyping

## Abstract

Acquisition of detailed anatomical and molecular knowledge from intact biological samples while preserving their native three-dimensional structure is still a challenging issue for imaging studies aiming to unravel a system’s functions. Three-dimensional micro-CT X-ray imaging with a high spatial resolution in minimally perturbed naive non-transparent samples has recently gained increased popularity and broad application in biomedical research. Here, we describe a novel X-ray-based methodology for analysis of *β-galactosidase* (*lacZ*) reporter-driven gene expression in an intact murine brain ex vivo by micro-CT. The method relies on detection of bromine molecules in the product of the enzymatic β-galactosidase reaction. Enhancement of the X-ray signal is observed specifically in the regions of the murine brain where expression of the *lacZ* reporter gene is also detected histologically. We performed quantitative analysis of the expression levels of *lacZ* reporter activity by relative radiodensity estimation of the β-galactosidase/X-gal precipitate in situ. To demonstrate the feasibility of the method, we performed expression analysis of the *Tsen54-lacZ* reporter gene in the murine brain in a semi-quantitative manner. Human mutations in the *Tsen54* gene cause pontocerebellar hypoplasia (PCH), a group of severe neurodegenerative disorders with both mental and motor deficits. Comparing relative levels of *Tsen54* gene expression, we demonstrate that the highest *Tsen54* expression is observed in anatomical brain substructures important for the normal motor and memory functions in mice.

## 1. Introduction

Molecular phenotyping of gene expression patterns without perturbing the three-dimensional (3D) cellular organization in tissue samples is a critical step for unraveling gene functions in normal physiology and disease [1]. Usually, histological analysis of gene expression on consecutive 2D sections using light or electron microscopy is the method of choice to accomplish such task. The 2D information is then digitally assembled into a 3D volume. This methodology suffers from substantial loss of volume information in terms of shape and morphology [2,3]. The application of sophisticated reconstruction methods is then required to correct a distorted 3D volume image obtained from a 2D stack. This computationally demanding step hampers an extensive implementation of the methodology both in research and clinical laboratories [4,5,6,7].

Innovative methodologies, such as two-photon microscopy, optical coherence tomography and confocal microscopy have been recently implemented for imaging of optically transparent tissues obtained by tissue clearing. Tissue clearing techniques make tissues transparent, thus reducing light scattering and absorption. This enables a deeper image acquisition, empowering the volumetric imaging of large-scale biological tissue samples, organs and even entire organisms [8,9,10,11,12,13,14,15,16,17]. The combination of tissue clearing with molecular labeling methods adds a cell specificity to whole organ and whole body imaging at cellular or subcellular resolution [18]. Despite the fact that tissue clearing methods are constantly evolving, they still suffer from several limitations. The small depth of light penetration through the samples requires the acquisition of images through multiple planes and, consequently, the necessity for the development and application of special algorithms and software in order to achieve correct 3D reconstructions. Sample treatment with harsh reagents might also lead to morphological changes within the anatomical structures [19,20]. 

Bioluminescence (BLI) is another optical imaging method applied for studies of tissues in normal development and during their pathological transformation [21,22]. It is based on monitoring of luciferase reporter gene expression in genetically modified cells or organisms. The luciferase reporter gene is expressed downstream of the target gene or promoter and detected after exogenous addition of luciferin substrates. The variety of engineered forms of luciferase genes from fireflies, beetles and marine organisms and many available substrates provides possibilities for simultaneous labeling of several genes [23]. Thus, it allows visualizing and quantifying molecular and cellular processes such as gene expression, protein–protein interactions, cell proliferation, migration and differentiation in different organs, including the brain, and in small animals in vivo [24]. The current limitation of BLI, however, is the low resolution, making it difficult to discriminate between transduced tissues. This limitation is currently being addressed, and promising studies to improve the resolution in 3D bioluminescence tomography have been recently presented [25].

Hard X-rays, which are characterized by high energy of >10 keV, have the potential to penetrate and image non-transparent thick specimens with high spatial resolution. As a result, X-ray analysis is an attractive and fast-developing method for non-invasive imaging of non-transparent tissues, organs and even whole organisms both in clinics and in research laboratories. At first, X-ray microtomography (micro-CT) imaging was used to analyze mineralized tissues which are characterized by high endogenous X-ray absorption or tissue radiodensity [26,27]. Imaging of soft organs and tissues by hard X-ray remains a challenging task due to the low endogenous radiodensity of soft tissues [28]. Treatment with contrasting stains containing high-atomic number elements and able to diffuse into tissues is necessary to increase the X-ray absorption properties of non-mineralized samples. At present, the most widely used contrasting agent is an iodine-based solution, I_2_KI or Lugol’s solution. The iodide and triiodide anions interact with (poly-)cationic molecules within the cells and increase the radiodensity of the tissue due to the high atomic number of iodine (Z = 65). Lugol’s solution became one of the most favored stains for X-ray imaging because of its ease of use, low cost and reversibility of the staining. This method was successfully applied for X-ray imaging of most organs and entire embryos of different model organisms [29,30,31]. Recently, eosin stain was implemented as an effective X-ray contrasting agent for ex vivo imaging of the murine kidney and brain. As with Lugol’s solution, negatively charged eosin molecules non-covalently interact with cationic amino acid side chains of proteins/peptides. The four bromine (Z = 35) atoms contained in the eosin molecule ensure the detectable increase in the radiodensity of stained tissues [32,33]. 

Several laboratories have demonstrated that fixed and dehydrated soft tissue samples embedded in paraffin, as routinely prepared for light microscopy–based histology, can be non-destructively imaged without the need for any contrasting stains either by conventional X-ray attenuation–based micro-CT or by X-ray phase-contrast synchrotron (XPCT) [34,35,36,37,38]. XPCT imaging allows increasing the resolution to the subcellular level with and without contrasting agents [39]. The main anatomical brain structures such as the cortex, hippocampus, thalamus, brain ventricular system and cerebellar layers (molecular, granular and Purkinje cell layers) as well as blood vessels can be visualized by X-ray and segmented based on their endogenous contrasting properties [39,40]. 

The advances in sample preparation methodologies proceed in parallel with technological developments in hard X-ray imaging modalities, which allow for imaging of biological samples with subcellular, approaching electron microscopy, resolution [39,40,41,42,43,44,45,46]. The next challenge is to add cell specificity potential to X-ray imaging procedures. This can be achieved by implementation of cell-specific labels with different X-ray-detectable radiodensities [47]. There are only a few successful examples of tissue-specific labeling with radiopaque probes published thus far. Two methods are based on metal detection of the peroxidase enzyme, conjugated to antibodies (metal immunodetection). Peroxidase reduces silver or nickel ions to an elemental state, resulting in precipitation of metal particles at labeled sites. The silver-based approach was used to visualize regions expressing acetylated-tubulin in the chick developing nervous system and type-II collagen in developing limbs by X-ray imaging [48]. The second is based on nickel-enhanced 3,3-diaminobenzidine (Ni-DAB) detection followed by staining with osmium tetraoxide and uranyl acetate to provide intrinsic contrast and enhance the DAB stain. Such protocol was applied for analysis of Ret expression in wild type and Ret mutant developing (E9,75) embryos [49]. The third report describes a genetic cell-specific targeting of the peroxidase reporter gene to the cellular compartments: peroxidase catalyzes the H_2_O_2_-dependent polymerization of 3,3′-diaminobenzidine (DAB) into a localized precipitate detectable by X-ray after treatment with OsO_4_. This gene reporter labeling scheme was used to enable X-ray imaging analysis of neuronal cell types and cellular substructures both in drosophila legs and in the murine brain [39].

A reporter gene assay, based on enzymatic features of a bacterial β-galactosidase (β-gal) enzyme encoded by the bacterial *lacZ* gene, was first described in the early 1980s [50]. Since then, *lacZ* is a very common gene reporter used for studies of cell-specific gene expression in bacteria, yeasts, mammalian cell lines and many model organisms. The *lacZ* reporter assay gained broad popularity in life science mainly due to the high stability of β-gal throughout histological fixation and tissue processing steps. Furthermore, enzymatic products of the β-gal reaction could be detected by a variety of substrates with specific chemical properties, allowing for a remarkable flexibility in the choice of detection methods. Both chromogenic and fluorogenic substrates to detect β-gal activity have been developed over the years and implemented for light-based imaging of different model organisms in vitro and in vivo [51,52]. Furthermore, *lacZ* reporter gene activity can be detected by magnetic resonance imaging (MRI). For this purpose, a gadolinium-based probe, EgadMe, was synthesized. β-gal cleaves this probe and releases the paramagnetic ion Gd3+, which, via direct interaction with water protons, increases the MR signal. This contrast agent was successfully used to follow *lacZ* expression in living *Xenopus laevis* embryos [53].

Among available β-gal substrates, 5-bromo-4-chloro-3-indolyl-β-D-galactoside (X-gal) is routinely used for reporter expression analysis. β-gal converts the X-gal substrate into 5,5′-dibromo-4,4′-dichloro-indigo, a blue stable and insoluble chromogenic product. This compound labels the cells expressing the promoter-driven *lacZ* reporter gene within tissues and organs, providing an effective anatomical and cell type-specific labeling of gene expression [54]. 

Pontocerebellar hypoplasia (PCH) is a heterogeneous group of autosomal recessive neurodegenerative disorders characterized by a wide diagnostic spectrum including delay in cognitive and motor development, seizures and death in early childhood. PCH is morphologically characterized by hypoplasia of the cerebellum and ventral pons, and progressive microcephaly with neocortical atrophy [55].

Genetic analysis of PCH patients identified several pathological mutations in a small numbers of genes. The majority of PCH cases have been linked to mutations in genes important for tRNA metabolism. In particular, 60% of all genetically defined cases of PCH are caused by mutations in the genes encoding for protein subunits of the TSEN complex [56,57,58,59,60]. The TSEN complex is composed of four subunits: TSEN54, TSEN34, TSEN15 and TSEN2. The main function of the complex is to remove introns from intron-containing pre-tRNA genes, a critical step for the production of functional tRNA [61]. Recessive mutations in all four subunits of the TSEN complex have been identified in PCH patients. Among these, mutations in the TSEN54 subunit account for 90% of all PCH patients with a mutation in the TSEN complex [57,58,59,62,63]. 

In the human developing brain, the *TSEN54* gene is highly expressed in the telencephalon, which gives rise to the cerebral cortex and basal ganglia within the cerebral hemispheres and metencephalon, which eventually forms the pons and cerebellum. *TSEN54* expression in the telencephalon and metencephalon was detected already at 8 weeks of gestation [62]. Later in development, at 23 weeks of gestation, a strong and specific expression of *TSEN54* in human cerebellar neurons has been reported [62]. While *TSEN54* mRNA has been extracted and detected in the adult human cortex and cerebellum (https://www.genecards.org/cgi-bin/carddisp.pl?gene=Tsen54 (accessed on 31 May 2021), and www.human.brain-map.org (accessed on 31 May 2021)), histological characterization of the *TSEN54* expression pattern in the adult brain was not performed. In model organisms, expression studies demonstrated that the *Tsen54* gene was ubiquitously expressed in the developing zebrafish (*Danio rerio*) [64]. In the murine brain, in situ hybridization data from the Allen Brain Atlas database suggest a very low, difficult to interpret signal (https://mouse.brain-map.org/experiment/show/69352788 (accessed on 31 May 2021).

Here, we developed a novel X-ray imaging-based analysis of gene expression of the *lacZ* reporter gene. We demonstrate that the product of the β-gal/X-gal reaction is a radiodense label for X-ray imaging of *lacZ* activity in situ. Our results show that the X-ray-detectable probes are the bromine atoms of the β-gal/X-gal reaction product. They produce an elevated local radiodensity within the tissues visualized as an increase in opaqueness. We applied this novel approach and evaluated its feasibility for the analysis of *Tsen54-lacZ* reporter gene activity in the murine brain. Using laboratory micro-CT, we analyzed murine brains expressing the *Tsen54-lacZ* reporter gene, previously stained with X-gal, dehydrated and embedded in paraffin. We observed that the combination of β-gal/X-gal staining and paraffin embedding of murine brains allows for both visualization and mapping of *lacZ* reporter expression to the anatomical brain substructures and regions. Furthermore, we demonstrate that density-based X-ray imaging allows performing a relative quantitative comparison of *lacZ* presence in different brain subregions within the same brain sample. Using the imaging platform presented here, we mapped and built a 3D view of the highest *Tsen54-lacZ* expression. These findings will allow focusing further studies on specific brain regions where the *Tsen54* gene is expressed with the aim to understand its function in normal development and in PCH disorder.

## 2. Results

### 2.1. Three-Dimensional Imaging of Tsen54-lacZ Reporter Gene Expression in Whole-Mount Mouse Brain by X-ray

First, we wanted to demonstrate the feasibility of X-ray micro-CT imaging for volumetric studies of gene expression. We analyzed the whole-mount mouse brains from the heterozygous *Tsen54-lacZ* (IKMC-*Tsen54^Tm1b/+^* line) animals. This reporter allele is also a knockout of the *Tsen54* gene (Supplementary Figure S1). We previously demonstrated that *Tsen54* is an essential gene. Homozygous *Tsen54-lacZ* animals die immediately at the post-implantation stage [30]. Heterozygous *Tsen54-lacZ* animals were analyzed by the International Mouse Phenotyping Consortium (IMPC). IMPC performs high-throughput phenotyping of mutant animals, assessing many physiological parameters (behavioral, metabolic, immunological, anatomical, etc.) with the goal to reveal as many phenotypes relevant to human disease as possible. The IMPC phenotyping data demonstrated that in the heterozygous *Tsen54-lacZ* animals, no pathological phenotypes were detected. The comprehensive IMPC phenotyping data can be found on the IMPC web page: (https://www.mousephenotype.org/data/genes/MGI:1923515 (accessed on 31 May 2021)). 

**Figure 1:**
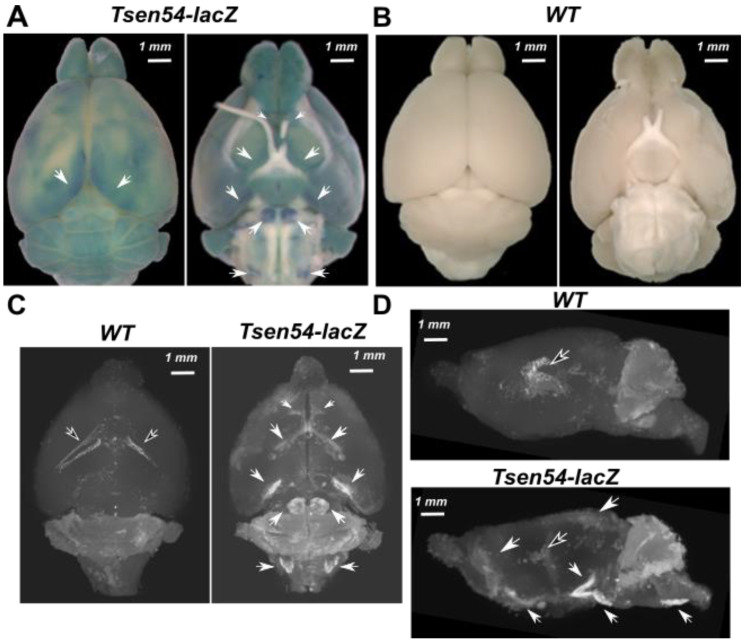
Three-dimensional imaging of *Tsen54-lacZ* expression in intact adult mouse brain by micro-CT. (**A**) Dorsal and ventral views of *Tsen54-lacZ* mouse brain stained with X-gal/FeCN and visualized by stereomicroscope; (**B**) dorsal and ventral views of littermate control whole mouse brain stained with X-gal/FeCN and visualized by stereomicroscope. (**C**) Representative maximum intensity projection (MIP) micro-CT images scanned with the resolution 5 μm/voxel of whole brain from *Tsen54-lacZ* (N = 3) and wild type *(WT)* (N = 3) animals. (**D**) Sagittal view of MIP images of the brains represented in panel (**C**). Additional regions of increased density (indicated by white arrows) in *Tsen54-LacZ* brains correspond to precipitated product of X-gal substrate converted by β-gal. White arrows indicate the regions of the elevated density in *Tsen54-lacZ* brains imaged by micro-CT and corresponding regions in the brains imaged by microscopy. The arrows filled with black indicate the endogenous densities detected both in the wild type and experimental brains.

We stained whole brains with X-gal to visualize the brain regions in which *lacZ* is expressed. The reaction was performed in the presence of potassium ferri- and ferro-cyanides, which are necessary as electron acceptors (X-gal/FeCN staining protocol) in the reaction [54]. The X-gal/FeCN staining’s blue precipitated product is strong and visible in brains from the *Tsen54-lacZ* mice, but not in wild type controls (Figure 1A,B).

We examined whether the deposited products of the β-gal reaction provide sufficient X-ray radiopacity. Hence, we performed micro-CT scans of the whole-mount X-gal/FeCN-stained, dehydrated and paraffin-embedded brains from *Tsen54-lacZ* and wild type animals. Micro-CT imaging detected the specific radiopaque regions of increased density in the *Tsen54-lacZ* brains, which were not observed in wild type controls (Figure 1C,D). Strikingly, our maximum intensity projection (MIP) of micro-CT analysis showed an overall good agreement with light stereomicroscopy images of the mouse brains (Figure 1A), both in frontal and sagittal 2D (Figure 1C,D) and in 3D views (Movies S1 and S2). This inspection revealed the differences in the distribution of X-ray-detected opaqueness when wild type and *Tsen54-lacZ* brains were compared. We hypothesize that additional regions of X-ray opaqueness in *Tsen54-lacZ* brains are due to X-ray detection of the lacZ/X-gal reaction product deposited in situ. 

### 2.2. Comparative Analysis of Virtual 2D Micro-CT and Histological Sections in the X-gal/FeCN Tained Brains

**Figure 2:**
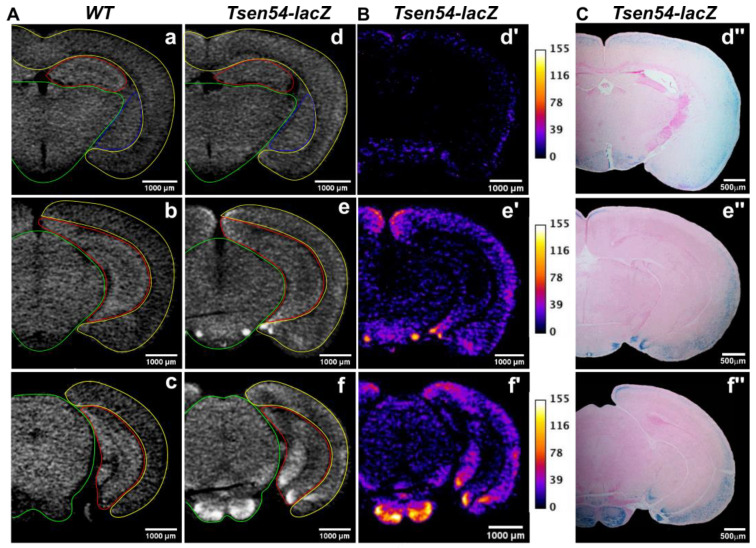
Comparison of virtual X-ray sections to histological sections of *Tsen54-lacZ* cerebrum reveals X-ray-detected regions of reporter gene expression. (**A**) Two-dimensional micro-CT-derived sections from cerebrum of wild type (**a**–**c**) and *Tsen54-lacZ* (**d**–**f**) animals. (**B**) Segmentation analysis of the virtual micro-CT-derived sections from *Tsen54-lacZ* brains (**d**′–**f**′). The lines delineate the segmentation regions: yellow—cortex; red—hippocampal plate; blue—amygdala; green—central brain. The Lookup Table (LUT) function was set from a 0 to 155 grayscale value interval. (**C**) Corresponding histological sections from the *Tsen54-lacZ* brain whole-mount stained with X-gal and counterstained with eosin solution (**d**″–**f**″). Micro-CT scans were performed with the resolution 20 μm/voxel.

The micro-CT image is a 3D matrix of density measurements, expressed in grayscale/brightness values calculated for every voxel. The volume image is digitally reconstructed from multiple 2D sections. Therefore, the brain volume can be computationally sliced, and corresponding 2D sections derived from different scans can be compared (Figure 2). To determine the differences in radiopaqueness between *Tsen54-lacZ* and wild type brains, we compared virtual 2D cerebrum sagittal sections from *Tsen54-lacZ* and wild type scans. Additional areas of high opaqueness in *Tsen54-lacZ* images absent in the wild type images were observed (Figure 2A). Then, we performed comparative quantitative analysis of *Tsen54-lacZ* images (Figure 2B). We subtracted the grayscale values calculated from wild type sections from the *Tsen54-lacZ* images using ImageJ. To compute this analysis, every image was segmented based on endogenous gray value differences in the following substructures: cortex, central brain region, hippocampus and amygdala. For each region, the mean and standard deviation of grayscale values were calculated separately. The sum of the mean and one standard deviation of wild type calculated grayscale values was set as the background correction threshold and subtracted from the mean grayscale values of every corresponding region of the *Tsen54-lacZ* image. The gray values obtained by this subtraction are a measure of the brightness differences between *Tsen54-lacZ* and analogues wild type 2D images (Figure 2B).

To demonstrate that the X-ray opaqueness is indeed produced by the β-gal reaction products deposited in situ, we performed a histological analysis of the *Tsen54-lacZ* brain sample. For this purpose, X-gal/FeCN-stained and paraffin-embedded brains, previously analyzed by micro-CT, were sectioned and reanalyzed by light microscopy (Figure 2C). We aligned corresponding 2D virtual micro-CT with histological sections. Our analysis demonstrated there is an agreement between areas having high X-ray opaqueness and those showing the presence of β-gal/X-gal/FeCN blue chromogenic precipitate (Figure 2B,C). Importantly, the histological analysis of wild type littermates’ brains did not show any chromogenic product of *lacZ* activity (data not shown) in agreement with previously published data [56]. We have to mention here that histological analysis provides cellular resolution, while the micro-CT analysis implemented here detects only the regions in which lacZ expression can be detected. This limitation can be overcome by using a synchrotron-based X-ray imaging methodology combined with *lacZ* reporter expression analysis.

**Figure 3:**
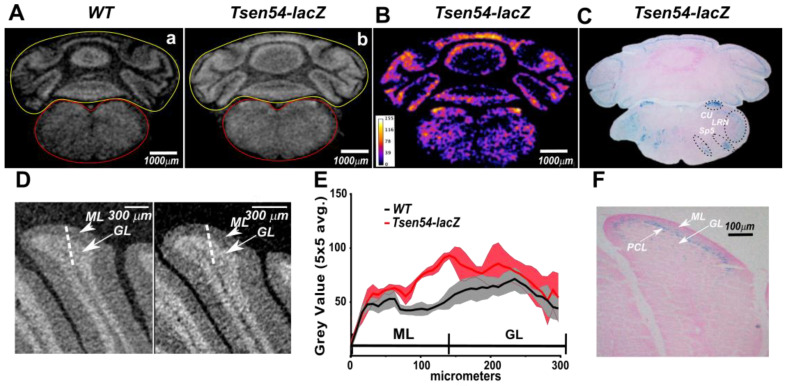
Comparative analysis of virtual X-ray and histological sections in hindbrain region. (**A**) Representative 2D virtual sections from hindbrain region of wild type (**a**) and *Tsen54-lacZ* (**b**) brains. Segmentation lines are indicated: yellow—cerebellar lobes, and red—brainstem. Resolution of the micro-CT scan is 20 μm/voxel. (**B**) Segmentation analysis of the 2D micro-CT hindbrain section. The LUT function was set from a 0 to 155 grayscale value interval. (**C**) Histological section of hindbrain from the *Tsen54-lacZ* brain. The regions with the highest expression in brainstem are indicated: CU—cuneate nucleus; Sp5—spinal nucleus of trigeminal; LRN—lateral reticular nucleus. (**D**) Two-dimensional micro-CT-acquired image of the simple cerebellar lobule from the *Tsen54-lacZ* and wild type animals at 5 μm/voxel resolution. The punctate lines indicate the regions selected for density quantification. Arrows indicate the cerebellar love layers. (**E**) Averages of brightness values, corrected for background, from *Tsen54-lacZ* (N = 3) and wild type (N = 3) cerebellar lobules, were plotted as a function of distance. (**F**) Histological analysis of the simple cerebellar lobule of cerebellum from the whole-mount X-gal/FeCN stained brains. ML—molecular layer; PCL—Purkinje cell layer; and GL—granular layer.

Next, we analyzed the 2D micro-CT sections from the hindbrain region (Figure 3A). The hindbrain was manually segmented in two subregions: (1) cerebellar lobes and (2) brainstem. We found that separate analyses of these two segments were necessary because of the endogenous substantial divergence of the medium gray values between the two hindbrain substructures. The background correction scheme described above was applied: the sum of the mean and one standard deviation of the wild type’s grayscale values of each of the two subregions was subtracted from the mean value of the corresponding subregion of the *Tsen54-lacZ* image. The background-corrected grayscale values are the X-ray-detected opaqueness above the background (Figure 3B). Comparison of this resulting image to the best corresponding histological section demonstrates that this high density results from in situ deposition of the β-gal/X-gal reaction product (Figure 3C).

To further verify that the increase in contrast produced by X-gal/FeCN staining is detected by X-ray, we focused on a smaller region within molecular and granular cerebellar layers (Figure 3D). To do this, we applied alternative experimental analysis. The regions of molecular and granular layers of the simple cerebellar lobe were compared using the Bruker CTAN program (Figure 3E). The gray pixel values were first corrected for the background caused by paraffin in which the samples were embedded. The average paraffin background gray value was calculated from a 300 μm-long stretch of pixels taken in an area occupied by paraffin only. In order to obtain background-corrected gray values for molecular and granular layers of the cerebellar lobe, we selected three parallel 300 μm-long and 1 voxel-wide stretches separated by 2 voxels (10 μm) crossing the boundary between molecular and granular layers (Supplementary Figure S1). The paraffin background pixel average value was subtracted from every pixel in each stretch, the mean and standard error were calculated for three related adjacent pixels in each line and final values were plotted (Figure 3E). This analysis demonstrated an increase in gray values in the molecular layer, with the maximum reached at the boundary between molecular and granular layers in the *Tsen54-lacZ* cerebellum. No differences in brightness between wild type and *Tsen54-lacZ* cerebellar lobes were observed in the granular layer (Figure 3E). The micro-CT grayscale values comparison is in agreement with the histological analysis showing that deposition of the chromogenic product of the β-gal/X-gal reaction occurs within the internal molecular layer and between molecular and granular layers (Figure 3F). In conclusion, our results demonstrate that the product of the biochemical β-gal/X-gal reaction performed in the presence of FeCN acceptors produces a radiodense, contrasting agent in situ detectable by X-ray imaging.

### 2.3. Relative Quantitative Analysis of the Tsen54-lacZ Gene Activity in Murine Brain

X-ray-based imaging has a unique feature among imaging techniques. X-ray imaging allows for a direct volumetric density quantification of an aggregated contrasting agent in situ. In our case, this means that the experimental densities are directly proportional to the *lacZ* reporter activity. The reconstructed X-ray brightness value at each voxel of the β-gal/X-gal-contrasted sample is the sum: (1) of the brightness derived from deposited products of the *β-gal* enzymatic reaction and (2) endogenous radiopaqueness of the dehydrated tissue. In this instance, the simple subtraction of the grayscale value of non-enhanced contrast tissue from contrast-enhanced tissue would return the absolute quantity of the deposited contrast agent at each voxel.

In this study, we used a Bruker Skyscan 1172 X-ray micro-CT system. To perform density comparison, we used calibrated datasets. Every dataset was calibrated using a phantom made of distilled water, with a known density of Hounsfield units (HU) = 0. The water sample was scanned under the same conditions used for brain samples, the mean grayscale value of the water sample was calculated and the brightness values of each scan were normalized as described in the Bruker manual (*Bruker-micro-CT Method Note: HU calibration, 2005*) both for *Tsen54-lacZ* and wild type brains. In this study, we adopted a relative comparison of brightness values within the same *Tsen54-lacZ* brain to evaluate the gene expression activity among different brain regions of the same dataset. 

**Figure 4:**
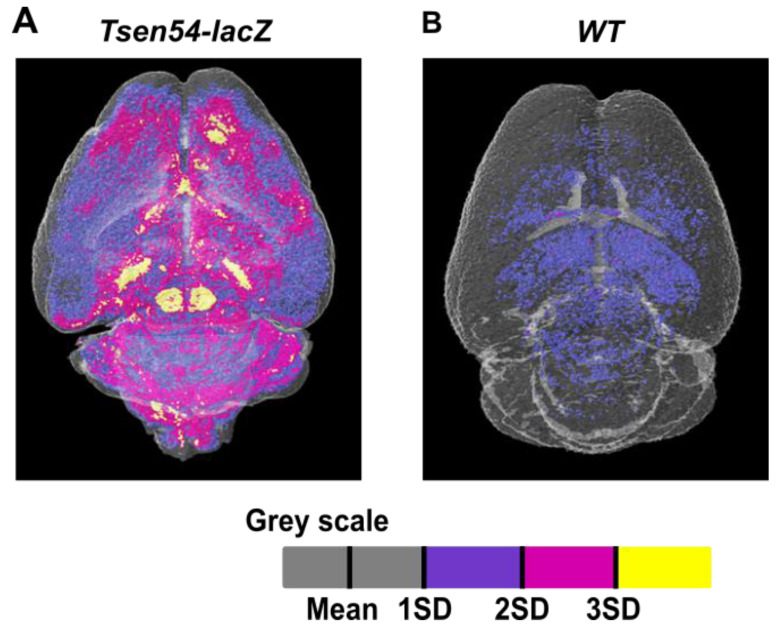
Relative quantification of *Tsen54-lacZ* gene expression by micro-CT analysis. (**A**,**B**) The average grayscale values were calculated using three datasets from the *Tsen54-lacZ* brains using CTAN (Bruker) software. The sum of the average mean and one standard deviation was set up as a background of non-contrasted tissues (gray), while values outside of this range were color-coded: 1SD(Standard Deviation) to 2SD—blue (lowest brightness); 2SD to 3SD—magenta (intermediate); and 3SD and above (maximum)—yellow. These calculated values were applied to the datasets obtained from the *Tsen54-lacZ* (**A**) and wild type (**B**) brains. Resolution of the micro-CT original images is 20 μm/voxel.

Using the calibrated datasets, we were able to perform relative quantitative comparison of the *Tsen54-lacZ* reporter activity. Furthermore, we conducted comparative analyses of densities’ distribution between wild type and experimental datasets. To accomplish the above tasks, the brains were first divided into two regions of interest (ROIs)—cerebrum (ROIc) and hindbrain (ROIh). Using CTAN Bruker software, means and standard deviations of gray value distributions for each ROI were calculated separately. The grayscale values of the paraffin background were excluded by applying the OTSU method (automatic image thresholding method) using Bruker software (CT-Analyser V1.13 UserManual). Then, the sum of the mean and one standard deviation calculated for the *Tsen54-lacZ* brain was defined as the threshold. The grayscale values above the threshold were subdivided into three grades: from 1SD to 2SD (low brightness values); 2SD to 3SD (intermediate); and from 3SD to maximum (highest). The two ROIs were treated independently and then assembled in a single volume image using Bruker CTVOL software (Figure 4A). The same values were also used for segmentation analysis of wild type brains (Figure 4B). Using this approach, we were able to perform a relative quantification of *Tsen54-lacZ* gene expression and classify the brain regions with high, medium and low activity of the reporter gene (Movie S3). These results demonstrate that the *lacZ* reporter enables identification of brain regions in which the *Tsen54* gene is expressed within X-ray images and allows for the relative comparison of the gene expression level in the specific brain regions.

### 2.4. Bromine Atoms in the β-gal/X-gal/FeCN Reaction Product Are the Radiodense Substrates for X-ray Imaging

The methodology presented here couples the chemical detection of β-*gal* enzymatic activity, that converts the X-gal substrate in chromogenic 5,5′-dibromo-4,4′-dichloro-indigo in the presence of ferri- and ferro-cyanides, with an in situ molecular signal detected as an increase in the X-ray attenuation coefficient within the brain. Thereby, the attenuation coefficients can be digitally converted into gray or brightness values. 

**Figure 5:**
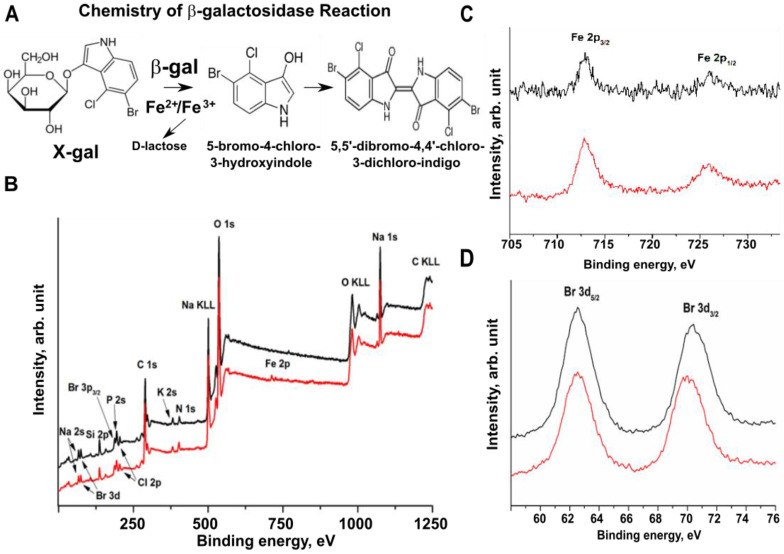
XPS analysis of the β-galactosidase enzymatic reaction products. (**A**) Schematic representation of the β-gal reaction in vitro. (**B**–**D**) X-ray photoemission spectroscopy (XPS) analysis of the β-gal reaction products: black lines—β-gal reaction performed with X-gal/FeCN substrate; red lines—X-gal/FeCN substrate only; (**B**) complete survey spectra of the β-gal reaction products; (**C**,**D**) regions of main photoemission spectra Fe 2p (**C**) and Br 3d (**D**) peaks; black lines—reaction of β-gal with X-gal/FeCN substrate; red lines—X-gal/FeCN substrate only. KLL- auger transition; β-gal-β-galactosidase.

We aimed to determine what molecular substrate is responsible for the radiodensity detected by X-ray imaging in our samples. The biochemistry of the β-ga/X-gal/FeCN reaction has been extensively studied since the early 1950s [65,66]. In brief, under proper conditions, the β-gal enzyme breaks the β-d-glycosidic linkage of X-gal, releasing a soluble, colorless indolyl monomer. Subsequently, two liberated indolyl moieties can form a dimer, if a proper acceptor, to which they can transfer an electron, is present in solution (Figure 5A). The ferric and ferrous ions are included in most *X-gal* reaction buffers exactly to provide the electron acceptor function to the solvent [67]. The above chemical steps produce a very stable, insoluble, bluish compound, which is an optically detectable β-gal reporter at the site of reaction. We wondered which compound, among the β-gal/X-gal reaction products, is able to adsorb the X-rays and acts as an X-ray reporter molecule in our experiments. The first hypothesis was that the presence of the electron transfer within the ferric/ferrous mix (FeCN) added to the X-gal reaction can lead to insoluble iron-containing products, such as FeIII, [FeIIIFeII(CN)6]3, similar to the reaction which occurs during Prussian blue synthesis [68]. We wondered if the hypothetical production of FeIII during the reaction could be responsible for the increase in X-ray absorption at the sites of the β-gal reaction.

To determine the molecules that could generate the high-contrast signal, we examined the atomic composition of the β-gal/X-gal/FeCN reaction products using X-ray photoemission spectroscopy (XPS). The atomic components of the reactions with and without pure β-gal in the presence of the X-gal/FeCN substrate were analyzed in vitro (Figure 5B–D). The XPS measurements excluded the presence of FeIII in the products of the β-gal reaction because Fe 2p3/2 peak at BE = 708.7 eV (Figure 5C) and there is absence of satellite peaks at a higher BE (lying at ~6 eV or ~8 eV from the main component), which are characteristic for FeII [69]. Indeed, no differences in the atomic composition between reactions performed in the presence of β-gal and control (substrate only) were detected.

**Figure 6:**
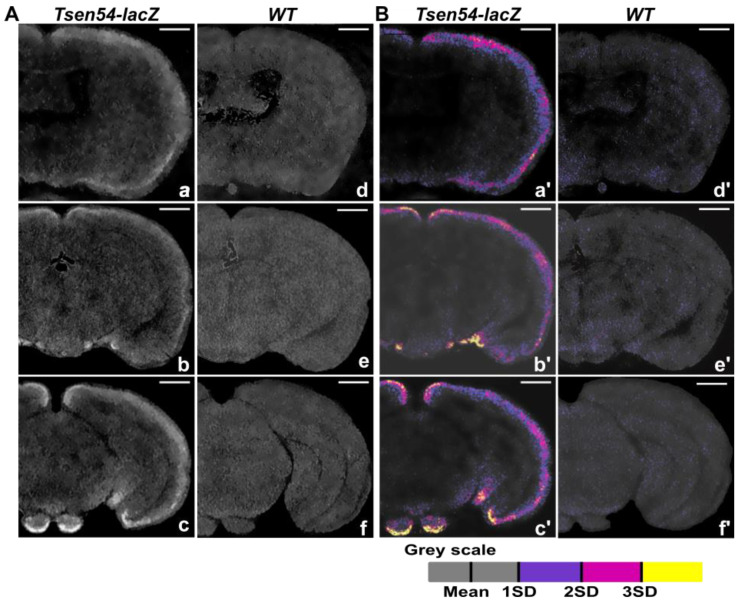
Virtual micro-CT sections from the *Tsen54-lacZ* murine cerebrum stained with X-gal in the presence of tetrazolium as an electron acceptor. (**A**) Virtual micro-CT sections of murine cerebrum from *Tsen54-lacZ* (**a**–**c**) and wild type (**d**–**f**) brains. Whole-mount brains were stained with X-gal in the presence of tetratzolium, and volume images were acquired with the resolution of 20 μm/voxel. (**B**) Segmentation analysis of the micro-CT-imaged brains was performed using CTAN (Bruker) software on 2D sections from *Tsen54-lacZ* (**a′**–**c′**) and wild type brains (**d′**–**f′**). Sum of mean and one standard deviation (1SD) calculated from an entire dataset was defined as a background (gray color); intervals between 1SD and 2SD—blue; 2SD and 3SD—magenta; and 3SD and maximum—yellow. The same values were applied for segmentation of the micro-CT-derived sections from the wild type brains. SD—standard deviation; Scale bar—1 mm.

To confirm this finding, we replaced FeCN solution with tetranitroblue tetrazolium chloride as an alternative electron acceptor substrate in the X-gal staining reaction. *Tsen54-lacZ* and wild type control brains were stained with X-gal/tetrazolium, dehydrated, embedded in paraffin and imaged by micro-CT (Figure 6). The datasets from *Tsen54-lacZ*/X-gal/tetrazolium-stained brains were calibrated using water as a phantom and segmented, using the CTAN program. The methodology for segmentation is described above. The grayscale values below the sum of the mean and 1SD were considered as a background, and values above were defined in three ranges: low from 1SD to 2SD; intermediate from 2SD to 3SD; and high above 3SD. We applied those calculations both for the analysis of virtual 2D micro-CT sections (Figure 6) and to the entire brain datasets (Movie S4). The comparison between the X-gal/tetrazolium and X-gal/FeCN methods demonstrated similar patterns of brightness distributions in *Tsen54-lacZ* brains. This finding holds true both for the regions in which *Tsen54-lacZ* gene expression was detected and for the relative quantitative estimation of intensities derived from reporter gene activity in specific brain areas (Movies S3 and S4). Importantly, the reproducible expression pattern of the *Tsen54-lacZ* reporter imaged by micro-CT was obtained irrespective of which staining methods and segmentation protocols were applied ( Figure 3; Figure 6, Movies S3 and S4). We conclude that the bromine atoms contained in the chromogenic product of the β-gal/X-gal reaction are in situ radiodense probes for X-ray imaging of *lacZ* reporter activity. Indeed, the mass attenuation coefficient of the Br atom at 40 kV (energy used in our micro-CT scan setting) is 6.832 μ/ρ, while the gray matter of the brain tissues is 25-fold lower and is 0.270 μ/ρ (https://www.nist.gov/pml/x-ray-mass-attenuation-coefficients (accessed on 31 May 2021)). This explains the elevated density detected by X-ray at the site of the 5,5′-bromo-4,4′-chloro-3-dichloro-indigo deposition in situ in the brain regions. To conclude, we demonstrate here that the β-gal/X-gal reaction product containing bromine atoms provides an in situ high-contrast enhancement measurable by X-ray imaging. 

### 2.5. Three-Dimensional Map of the Brain Regions Characterized by Specific Tsen54-lacZ Reporter Gene Expression

Based on the expression data presented in this manuscript, we performed a mapping of the brain regions with established *Tsen54-lacZ* expression. The 3D view of *Tsen54-lacZ* reporter expression was manually built by analyzing both 3D and 2D micro-CT imaging modalities and histological sections. The regions with *Tsen54-lacZ* expression were determined using the 2D and 3D Allen Brain Virtual Reference Atlas tool (http://atlas.brain-map.org/ (accessed on 31 May 2021)) (Movie S5). The anatomical structures characterized by the highest and intermediate levels of *Tsen54* expression are summarized in Table 1.

## 3. Discussion

In this manuscript, we introduced a novel methodology for gene expression analysis of whole-mount murine brains. We validated it by studying the expression of *Tsen54*, a gene causing the rare disease pontocerebellar hypoplasia, in the mouse brain. This methodology is based on using the gene promoter of interest driving the *lacZ* reporter and conventional laboratory micro-CT X-ray imaging equipment. We demonstrated that the non-soluble precipitate product of β-gal/X-gal deposited in situ of the enzymatic reaction is a radiodense probe for X-ray detection of β-gal activity. 

A key feature of this micro-CT method is that it allows for volumetric localization of the specific anatomical structures and subregions in which the reporter gene is expressed. This is possible because we combined the X-gal staining protocol with dehydration by ethanol and paraffin embedding of the mouse brain. The dehydration process, by removing water molecules, increases the tissue density and renders the mouse brain visible to hard X-ray imaging without the need for any additional treatment with non-specific contrasting agents. In addition, the paraffin embedding maintains the shape and correct morphology of both the whole sample and internal brain structures. By combining this sample preparation with X-gal staining, we obtained micro-CT images of murine brains exhibiting clear views of both anatomical brain substructures and radiodense *lacZ* gene expression sites. We managed to correlate them with the stereotaxic coordinate of the mouse brain atlas ([70] and https://mouse.brainmap.org (accessed on 31 May 2021)). We performed manual registration analysis of the *Tsen54-lacZ* expression using the reference Mouse Alan Brain Atlas. The process of registration can be substantially improved by applying advanced bioinformatics protocols based on machine learning algorithms, which will allow for automatic recognition of anatomic regions where the *lacZ* reporter gene is expressed. Such program for automatic anatomical brain region registration has been recently presented [71]. 

The X-ray imaging modality does not damage the sample and allows a consecutive histological analysis. The samples after X-ray imaging can be sectioned and further investigated by light microscopy. Thus, the application of micro-CT imaging inverts the sequence of steps usually performed to obtain 3D images from histological consecutive 2D sections because the brain samples are first imaged and then analyzed histologically. This combined imaging pipeline is optimized to obtain the most out of the functional data from biological samples. 

A very practical advantage of the method is based on the usage of the low-cost X-gal substrate for detection of β-gal activity and the procurability of *lacZ* reporter-expressing model organisms. Over the years, many *lacZ* reporter murine lines were engineered both in single laboratories and as part of global projects, such as the International Knockout Mouse Consortium (IKMC). In our studies, we took advantage of an available reporter/knockout *Tsen54-lacZ* murine line engineered by IKMC for the International Mouse Phenotyping Consortium (IMPC). The IMPC has the goal to determine the functions of every mammalian gene by systematic physiological and molecular phenotyping of 20,000 knockout mouse lines. These knockout alleles are engineered by replacing a critical gene exon with the *lacZ* reporter gene. Therefore, all knockout murine lines are *lacZ* reporter lines (https://www.beta.mousephenotype.org/about-ikmc-strategies (accessed on 31 May 2021)) [72]. To date, about 5000 *lacZ* reporter mouse lines have been produced by the Consortium (www.mousephenotype.org (accessed on 31 May 2021)) and are available to the scientific community [73]. The data accumulated by the IMPC have demonstrated that *lacZ* is an excellent reporter system to study gene expression in ex vivo murine organs because it faithfully recapitulates the activity of the promoter that drives the *lacZ* gene. In addition, it has been demonstrated that endogenous *lacZ* activity in the mouse brain does not produce a detectable background in light imaging studies [54]. Additionally, the *lacZ* gene reporter can be delivered in a tissue-specific manner in the brains of wild type animals without the need for tissue-specific germ line reporter targeting by gene delivery methods, such as adenoviral infection and electroporation [74,75]. 

We performed our analysis of the murine brains using top-bench Bruker Skyscan 1172 micro-CT that has a resolution ranging from 5 to 20 μm/voxel. This resolution permits inspecting, in detail, the anatomical substructures in which the reporter gene is expressed but not high enough to identify the cell types expressing *lacZ*. This obstacle can be overcome by using more advanced micro-CT apparatuses, which support a substantial increase in imaging resolution such as nano-CT systems, propagation-based X-ray phase-contrast tomography and synchrotron-based X-ray microtomography. Several groups demonstrated the feasibility of these advanced X-ray-based technologies for the analysis of murine organs and, specifically, the mouse brain at cellular resolution, as such making the resolution of X-ray imaging methods comparable to histological analysis [39,40,41,42]. The application of these up-to-date machineries to our X-ray-based gene expression method would be highly beneficial to directly link gene expression patterns with cellular specificity in three dimensions.

A feature that is unique to the X-ray imaging procedure, which gives it an unbiased strength for gene expression analysis, is that it is based on the detection of the density of an aggregated precipitate produced by the enzymatic β-gal/X-gal reaction. Thereby, it enables a way for both relative and absolute quantitative analysis of the promoter activity which drives the *lacZ* reporter. Hence, we introduced, here, a scheme aimed at the relative quantitative estimation of *Tsen54-lacZ* reporter gene expression. This analysis is based on comparison of grayscale values in different anatomical brain substructures within the same micro-CT dataset. This approach, however, does not allow for evaluation of gray values on an absolute scale and for direct comparison between different datasets because of the current limitations of scanning calibration methods. Yet, there is a huge potential to achieve the absolute quantification of the densities derived from the *lacZ* gene activity by developing novel instrument calibration procedures. In our experiments, we conducted density-based calibration of images using water as a phantom, a common standard for normalization of datasets. For future studies, the calibration phantoms of the lacZ/X-gal product obtained after incubation with a known amount of the β-gal enzyme could be custom-built. With this calibration scheme in hand, the absolute quantification of lacZ activity within the samples and their comparison will be feasible.

In addition, β-gal labeling can be combined with existing metal-based X-ray labels in order to achieve simultaneous cell type-specific labeling in the same volume image. This would not only empower X-ray analysis of soft tissues to a level analogous to cell-specific immune-fluorescence imaging but also would provide the option to perform an absolute quantitative analysis of the labeled targets.

The data presented by us demonstrate that the β-gal reaction product has radiodense characteristics ex vivo. This knowledge can also lead to the development of novel β-gal-based molecular probes for in vivo X-ray-based imaging of gene expression. The main obstacle to use X-gal as a substrate for in vivo imaging is that X-gal does not diffuse inside living mammalian cells. Therefore, possible chemical modification of the X-gal molecule, which would lead to diffusion of the X-gal substrate across the cell membrane in live cells, will allow extending X-ray imaging of *lacZ* expression analysis to living organisms. This would make in vivo X-ray-based *lacZ* reporter analysis similar to quantitative bioluminescence imaging with the addition of tomographic characteristics. This possibility would provide important knowledge of the modulation of gene activity in development and in response to environmental events in normal model organisms and in disease.

Our micro-CT-based analysis demonstrated that the *Tsen54-lacZ* reporter is diffusely expressed in the cortex, with the highest expression observed in the retrosplenial cortex (RSC) which is important for a variety of cognitive tasks including memory, navigation and prospective thinking both in rodents and in mice [76,77]. This suggests that *Tsen54* may play an important role in cognitive processes and, specifically, in the formation of spatial memory in humans and mice.

High *Tsen54* expression is observed in the temporal lobe of the dentate gyrus, the region which is essential for declarative memory (conscious memory for facts and events) and spatial memories in humans and in rodents [78]. Very specific high *Tsen54* expression marks the lateral mammillary (LM) nucleus. The LM nucleus is part of the hypothalamic system of the interbrain. Lateral mammillary bodies are critical for generating signals controlling head direction and movements [79]. In the cortical plate of the brain, a high-expression pattern forms a fork-like structure at the rostral part of the murine brain, which is composed of several substructures: the anterior olfactory nucleus, the bed nucleus of the accessory olfactory (BA), the nucleus of the lateral olfactory tract (NLOT), the lateral preoptic area tract (LPO) and the vascular organ of the lamina terminals. 

Especially interesting is an expression pattern observed in the hindbrain. Particularly strong signals were detected in the pons, with the highest expression limited to the pontine gray area. These data suggest an important role for the *Tsen54* gene specifically in development and functions of the pontine area and are also supported by the knowledge that in PCH patients, a variable degree of pons flattening is observed [59]. In the medulla, high expression in precerebellar nuclei: external cuneate, lateral reticular and spinal trigeminal, is observed. These pons and medulla substructures are important for proper signal exchange with the cerebellum, exerting fine control on balance, motor movements and motor learning [80]. All these results demonstrate that the function of the *Tsen54* gene is strictly required especially in the regions critical for memory processing and body movement control.

In summary, here, we introduced a novel method for 3D characterization of *lacZ* reporter gene expression by X-ray micro-CT imaging. The presented method provides research with an additional tool for relative comparison of gene expression within an entire organ. This method can be combined with histological and light microscopy analysis and could provide a quantitative dimension to the analysis of lacZ reporter gene activity. 

## 4. Material and Methods

### 4.1. Ethics Statement

After weaning, mice were housed by litters of the same sex, 3 to 5 per cage, and maintained in a temperature-controlled room at 21 ± 2 °C, on a 12-h light/dark cycle (lights on at 7 a.m. and off at 7 p.m.), with food and water available ad libitum in a specific pathogen-free facility. The experimental protocols and animal care procedures were reviewed and approved by the Ethical and Scientific Commission of Veterinary Health and Welfare Department of the Italian Ministry of Health (protocol approval reference: 118/2012-B). Approval is in agreement with the Italian laws and regulations according to the ethical and safety rules and guidelines for the use of animals in biomedical research in application of the relevant European Union’s directives (n. 86/609/EEC and 2010/63/EU).

### 4.2. Animals

The *Tsen54-lacZ* reporter allele was engineered by the IKMC. The targeting strategy is deposited in the IMPC portal (https//www.mousephenotype.org (accessed on 31 May 2021)). In brief, knockout mice were generated from *Tsen54^tm1a(EUCOMM)Wtsi^* ES cells (https://www.mousephenotype.org/data/genes/MGI:1923515 (accessed on 31 May 2021)) and are depicted in Supplementary Figure S2. ES cells have a C57Bl/6N background. The ES cells were injected in blastocysts, and germ line transmission was obtained from chimeras. All animals were maintained only on C57Bl/6N background for all generations. To remove the *neo* selection cassette, the *Tsen54^Tm1a (EUCOMM)Wtsi^* animals were crossed with *ROSA26Cre* (MGI: 5285392; C57Bl/6N background) animals. As a result, heterozygous animals with the *Tsen54^Tm1b^* allele were produced. The *Tsen54^Tm1b^* allele is a knockout and *lacZ* reporter allele of the *Tsen54* gene in which exon 6 is replaced by the *lacZ* reporter (Supplementary Figure S2). A total of 10 mouse brains were used for analyses. The brains were obtained from littermates (5 mice of *Tsen54-lacZ* and 5 wild type animals).

### 4.3. Processing for Micro-CT

Prior to perfusion, adult animals (at least 2 months old) were anesthetized with 2.5% avertin solution (100 μL/10 g of body weight). The animals then were intracardially perfused with PBS (20 mL per animal at room temperature) followed by chilled 4% PFA (25 mL per animal). Brains were extracted from the skull and fixed for an additional 30 min with 4% PFA on ice. The brains were then incubated in wash buffer (2 mM MgCl_2_, 0.01% sodium deoxycholate, 0.02% of Nonidet-P40, 0.1 M sodium phosphate buffer pH 7.3) for 30 min on ice. Brains were stained with 1 mg/mL X-gal, freshly added from stock solution of 25 mg/mL X-gal (Sigma-Aldrich, B4252) in dimethylformamide, to wash buffer supplemented with 0.2% of potassium ferrocyanide (Sigma-Aldrich, P-9387) and 0.16% potassium ferricyanide (Sigma-Aldrich, P-8131) for 48 h at 37 °C. For staining in the presence of tetranitroblue tetrazolium chloride instead of potassium ferrocyanide and potassium ferricyanide, tetranitroblue tetrazolium chloride (Sigma cat: 87961) was added to wash buffer at a final concentration of 3.3 μg/mL. After staining, brains were rinsed in wash buffer and additionally fixed with 4% PFA, overnight in the cold room. The embedding was performed as described [81]. Briefly, samples were dehydrated by incubation in graded series of ethanol solution: 50% ethanol for 2 h, 70% ethanol overnight and 95% ethanol for 1 h. All steps were performed in the cold room. Dehydration of the brains was completed by incubation in 100% ethanol at room temperature for 2 h. Subsequently, the samples were transferred in xylene and incubated for 45 min. Then, the samples were transferred in xylene/paraffin mixture (1:1 ratio) and incubated at 56 °C for 45 min. After incubation was completed, samples were placed in fresh liquid paraffin and incubated overnight at 56 °C. The next morning, the paraffin was replaced with fresh paraffin, and samples were incubated for 1 h at 56 °C. Then, the brains were transferred in the embedding molds filed with liquid paraffin and left to polymerize for 1 h at room temperature.

### 4.4. Micro-CT Imaging

Images were acquired using Skyscan 1172G (Bruker, Kontich-Belgium) with an L7901-20 Microfocus X-ray Source (Hamamatsu). The paraffin-embedded brains were attached using Orthodontic Tray Wax (09246 Kerr) to the micro-CT imaging platform and placed vertically inside the instrument. The X-ray source was set at 39 kV and the current at 240 µA (9 W). Scans were performed with 650 and 700 projections at full rotation, with a total scan time of approximately 30 min. The following scanning parameters were applied: no filter, near camera position, dimension: 1000 × 666 (1 K), large pixel size, exposure time 60 ms, magnification 20 µm, rotation step 0.4° and partial width 88%. Single images (stored as TIFF-file) were reconstructed from projection images using NRecon Skyscan Reconstruction software (Brucker). The following reconstruction parameters were applied: smoothing set at 2, misalignment compensation at +/−2, ring artefact reduction at 6, beam-hardening correction at 40%.

The reconstructed datasets were calibrated in Hounsfield units (HU), using water as a phantom and CTAN software. The calibration was performed as described in the Bruker manual (*Bruker-micro-CT Method Note: HU calibration, 2005)*. After calibration, water HU = 0 and air HU = −1000 were implemented. The datasets were analyzed using analyzer software CTAN v 1.13 (Bruker) and CTVOL 2.0 and CTVOX (Bruker) software. To obtain volume rendering views and 3D movies, visualization software CTVOL v 2.0 was used. Multiple intensity projection analysis was performed using CTVOX (Bruker) software.

### 4.5. Light Microscopy

X-gal/FeCN-stained brains, embedded in paraffin, were sectioned at 16 μm and stained with eosinY 1% aqueous solution (Bio-Optica 05-M10002). Images were obtained with the stereomicroscope MZ12 (Leica) equipped with a color camera.

### 4.6. XPS Analysis of β-Galactosidase Reaction Products In Vitro

The reaction was performed in 50 μL volume of wash buffer with 0.2% of potassium ferrocyanide (Sigma-Aldrich, P-9387) and 0.16% potassium ferricyanide (Sigma-Aldrich, P-8131) supplemented with 1 mg/mL of X-gal, freshly added from stock solution (25 mg/mL X-gal (Sigma-Aldrich, B4252) in dimethylformamide) with 0.16 units of β-galactosidase (Sigma cat: G4155) or without an enzyme as a control. Then, the entire volumes were deposited on the glass as a thin film and dried at room temperature. XPS measurements were carried out in a spectrometer Escalab MkII (VG Scientific Ltd., London, UK) by using an Al Kα source. The acquisition parameters were the following: analyzer pass energy = 40 eV, and large area lens mode A1 × 22 with an analysis diameter of about 10 mm.

## Figures and Tables

**Table 1:** Summary of the anatomical substructures characterized by a medium to high relative expression level of *Tsen54-lacZ* gene reporter. Anatomical brain structures and substructures characterized by high and intermediate relative expression level of *Tsen54-lacZ* reporter gene are listed. The color code for the indicated regions is extrapolated from Allen Brain Atlas BrainExplorer 2 software. This color code was applied for virtual reconstruction of 3D *Tsen54* gene expression pattern in the murine brain (Movie S5); REL—relative expression level of the reporter gene activity. Yellow indicates areas of subregions with highest and magenta with intermediate gene expression levels.

Anatomical Region	Anatomical Structure	Anatomical Sub-Structure	Color Code	REL	
Cortical plate	Isocortex	Secondary motor area Layer 1	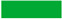	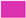	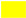
Cortical plate	Isocortex	Retrosplenial area, dorsal part	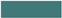	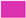	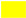
Cortical plate	Isocortex	Perirhinal area, Layer 1	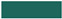	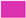	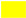
Cortical plate	Hippocampal formation	Dentate gyrus, Molecular cell layer (temporal lobe)		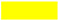	
Cortical plate	Olfactory area	Nucleus of the lateral olfactory tract (NLOT)	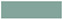	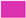	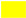
Cortical plate	Olfactory area	Anterior olfactory nucleus (AON)	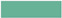	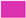	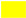
Cerebral nuclei	Striatum	Bed nucleus of the accessory olfactory tract (BA)	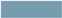	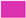	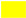
Interbrain	Hypothalamus	Lateral mammilary nucleus (LM)	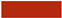	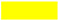	
Interbrain	Hypothalamus	Lateral preoptic area (LPO)	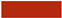	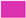	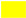
Hindbrain	Medulla (Motor related)	Lateral reticular nucleus (LRN)	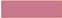	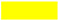	
Hindbrain	Medulla (Sensory related)	Cuneate nucleus (CU)	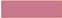	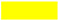	
Hindbrain	Medulla (Sensory related)	Spinal nucleus of trigeminal caudal part	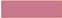	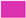	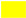
Hindbrain	Pons (Motor related)	Pontine gray	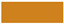	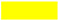	
Cerebellum	Cerebral cortex	Vermal regions	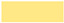	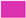	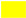
Cerebellum	Cerebral cortex	Hemispheric regions	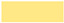	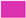	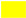

## Data Availability

Not applicable.

## Supplementary Materials

The following are available online at https://www.mdpi.com/article/10.3390/brainsci11060746/s1, Figure S1: MicroCT images of cerebellar lobe from wild type (A) and *Tsen54-lacZ*(B) brains stained with FeCH and imaged with the resolution of 5 μm/voxel. The 300 μm lines indicate the region from which paraffin background grey values calculations were performed. Three parallel 300 μm lines separated by 2 pixels were drown through molecular and granular layers. Three voxels were averaged and mean and standard deviation were calculated. The background grey values were subtracted from every averaged pixels. Distribution of grey scale intensity values were plotted in Figure 3E; Figure S2: A.Schematic representation of the *Tsen54^tm1a^* allele. *lacZ* reporter gene is inserted into intronic locus, following exon5, replacing exons 6 of the *Tsen54* gene. *Cre*-mediated recombination removes exon6 and neo targeting cassette from genomic *Tsen54* gene locus and convert *Tsen54^tm1a^* allele into *Tsen54^tm1b^* allele. B. The *Tsen54^tm1b^* allele is a knockout of *Tsen54* gene and is a *lacZ* reporter allele of *Tsen54* gene expression. *Ex*- exon; *En2A SA*-splice acceptor site; *IRES*-internal ribosomal entry site; *lacZ* - bacterial *β**-galactosidase* reporter gene; *pA*-poly A; *hBactP*- human *β*-actin promoter; *neo*-neomycin resistance gene; *FRT- FLP* recombination sites and *LoxP*–*Cre* recombination sites are indicated; Video S1: MicroCT derived RGB volume of the *Tsen54-lacZ* mouse brain stained with X-gal/FeCN method. The volume image was acquired using camera resolution of 1 K (image matrix of 1000 × 575 pixel with the 20 μm/voxel size). Screenshot of the transfer function editor windows of the CTVOX analyser (Bruker software) demonstrates setting of the RGB transfer function curves for building a colour volume-rendered 3D model; colour coded for the tissue density function: red-blue-green; transparency level defined by the purple line (supplementary info); Video S2: MicroCT derived RGB movie of the wild type mouse brain stained with X-gal/FeCN method. The volume image was acquired using camera resolution of 1 K (image matrix of 1000 × 575 pixel with the 20 μm/voxel size). Screenshot of the transfer function editor windows of the CTVOX analyser (Bruker software) demonstrates setting of the RGB transfer function curves for building a colour volume-rendered 3D model; colour coded for the tissue density function: red-blue-green; transparency level defined by the purple line (supplementary info); Video S3: Segmantation analysis of the *Tsen54-lacZ* brain, whole-mount stained with X-gal/FeCN and imaged by microCT. MicroCT imaging was performed with the resolution of 20 m of voxel size. Calculated using CTAN (Bruker software) mean of Hounsfield units from the *Tsen54-lacZ* brain and one standard deviation (1SD) were defined as a background, while values outside of this range were colour coded: values from 1SD to 2SD-blue; from 2SD-3SD-magenta; from 3SD and up to maximum–yellow. The reconstruction of the segmented *Tsen54-lacZ* brain was performed using CTVOL analysis (Bruker software); Video S4: Segmentation analysis of the *Tsen54-lacZ* brain whole-mount stained with X-gal/tetrazolium protocol and imaged by microCT with the resolution of 20 μm of voxel size. Calculated using CTAN (Bruker software) mean of Hounsfield units of the *Tsen54-lacZ* brain and one standard deviation (1SD) were defined as a background, while values outside of this range were colour coded: values from 1SD to 2SD-blue; from 2SD-3SD-magenta; from 3SD and up to maximum–yellow. The reconstruction of the segmented *Tsen54-lacZ* brain was performed using CTVOL analysis (Bruker software); Video S5: Virtually reconstructed in three-dimension expression pattern of *Tsen54* gene. The reconstruction was based on the microCT analysis of the *Tsen54-lacZ* mouse brain ex vivo and performed using A. Brain Atlas BrainExplorer2 software using Allen Mouse Common Coordinate Framework option. The selected for the 3D reconstruction brain structures are listed in Table 1. Dentate gyrus was not included because expression was observed only in the temporal lobe of dentate gyrus. Cerebellum also was excluded from virtual reconstruction for the best visualization of the nuclei in medulla.

## References

[1] Streicher J., Donat M.A., Strauss B., Spörle R., Schughart K., Müller G.B. (2000). Computer-based three-dimensional visualization of developmental gene expression. Nat. Genet..

[2] Lein E.S., Hawrylycz M.J., Ao N., Ayres M., Bensinger A., Bernard A., Boe A.F., Boguski M.S., Brockway K.S., Byrnes E.J. (2007). Genome-wide atlas of gene expression in the adult mouse brain. Nature.

[3] Knowles D.W., Biggin M.D. (2013). Building quantitative, three-dimensional atlases of gene expression and morphology at cellular resolution. Wiley Interdiscip Rev. Dev. Biol..

[4] Ju T., Warren J., Carson J., Bello M., Kakadiaris I., Chiu W., Thaller C., Eichele G. (2006). 3D volume reconstruction of a mouse brain from histological sections using warp filtering. J. Neurosci. Methods.

[5] Brandle K. (1989). A new method for aligning histological serial sections for three-dimensional reconstruction. Comput. Biomed. Res..

[6] Weninger W.J., Streicher J., Muller G.B. (1996). 3-dimensional reconstruction of histological serial sections using a computer. Wien. Klin. Wochenschr..

[7] Weninger W.J., Meng S., Streicher J., Müller G.B. (1998). A new episcopic method for rapid 3-D reconstruction: Applications in anatomy and embryology. Anat. Embryol..

[8] Denk W., Strickler J.H., Webb W.W. (1990). Two-photon laser scanning fluorescence microscopy. Science.

[9] Helmchen F., Denk W. (2005). Deep tissue two-photon microscopy. Nat. Methods.

[10] Micheva K.D., Smith S.J. (2007). Array tomography: A new tool for imaging the molecular architecture and ultrastructure of neural circuits. Neuron.

[11] Li A., Gong H., Zhang B., Wang Q., Yan C., Wu J., Liu Q., Zeng S., Luo Q. (2010). Micro-optical sectioning tomography to obtain a high-resolution atlas of the mouse brain. Science.

[12] Quintana L., Sharpe J. (2011). Optical projection tomography of vertebrate embryo development. Cold Spring Harb Protoc..

[13] Botcherby E.J., Smith C.W., Kohl M.M., Debàrre D., Booth M.J., Juškaitis R., Paulsen O., Wilson T. (2012). Aberration-free three-dimensional multiphoton imaging of neuronal activity at kHz rates. Proc. Natl. Acad. Sci. USA.

[14] Kuwajima T., Sitko A.A., Bhansali P., Jurgens C., Guido W., Mason C. (2013). ClearT: A detergent- and solvent-free clearing method for neuronal and non-neuronal tissue. Development.

[15] Chung K., Wallace J., Kim S.Y., Kalyanasundaram S., Andalman A.S., Davidson T.J., Mizabekov J., Zalocusky K.A., Mattis J., Denisin A.K. (2013). Structural and molecular interrogation of intact biological systems. Nature.

[16] Ke M.T., Fujimoto S., Imai T. (2013). SeeDB: A simple and morphology-preserving optical clearing agent for neuronal circuit reconstruction. Nat. Neurosci..

[17] Susaki E.A., Tainaka K., Perrin D., Kishino F., Tawara T., Watanabe T.M., Yokoyama C., Onoe H., Eguchi M., Yamaguchi S. (2014). Whole-brain imaging with single-cell resolution using chemical cocktails and computational analysis. Cell.

[18] Tomer R., Lovett-Barron M., Kauvar I., Andalman A., Burns V.M., Sankaran S., Grosenick L., Broxton M., Yang S., Deisseroth K. (2015). SPED light sheet microscopy: Fast mapping of biological system structure and function. Cell.

[19] Tian T., Yang Z., Li X. (2020). Tissue clearing technique: Recent progress and biomedical applications. Review. J. Anat..

[20] Gómez-Gaviro M.V., Sanderson D., Ripoll J., Desco M. (2020). Biomedical applications of tissue clearing and three-dimensional imaging in health and disease. iScience.

[21] Concilio S.C., Russell S.J., Peng K.W. (2021). A brief review of reporter gene imaging in oncolytic virotherapy and gene therapy. Mol. Ther. Oncolytics.

[22] Mezzanotte L., van’t Root M., Karatas H., Goun E.A., Löwik C. (2017). In vivo molecular bioluminescence imaging: New tools and applications. Trends Biotechnol..

[23] Branchini B.R., Southworth T.L., Fontaine D.M., Kohrt D., Florentine C.M., Grossel M.J. (2018). A firefly luciferase dual color bioluminescence reporter assay using two substrates to simultaneously monitor two gene expression events. Sci. Rep..

[24] Nemes B., Bölcskei K., Kecskés A., Kormos V., Gaszner B., Aczél T., Hegedüs D., Pintér E., Helyes Z., Sándor Z. (2021). Human Somatostatin SST 4 Receptor Transgenic Mice: Construction and Brain Expression Pattern Characterization. Int. J. Mol. Sci..

[25] Hall M.P., Woodroofe C.C., Wood M.G., Que I., Van’t Root M., Shi C., Kirkland T.A., Encell L.P., Wood K.V., Löwik C. (2018). Click beetle luciferase mutant and near infrared naphthyl-luciferins for improved bioluminescence imaging. Nat. Commun..

[26] Neues F., Goerlich R., Renn J., Beckmann F., Epple M. (2007). Skeletal deformations in medaka (Oryzias latipes) visualized by synchrotron radiation micro-computer tomography (SRmicroCT). J. Struct. Biol..

[27] Neues F., Epple M. (2008). X-ray microcomputer tomography for the study of biomineralized endo- and exoskeletons of animals. Chem. Rev..

[28] Saito S., Murase K. (2012). Ex vivo imaging of mouse brain using micro-CT with non-ionic iodinated contrast agent: A comparison with myelin staining. Br. J. Radiol..

[29] Wong M.D., Spring S., Henkelman R.M. (2014). Structural stabilization of tissue for embryo phenotyping using micro-CT with iodine staining. PLoS ONE.

[30] Ermakova O., Orsini T., Gambadoro A., Chiani F., Tocchini-Valentini G.P. (2018). Three-dimensional microCT imaging of murine embryonicdevelopment from immediate post-implantation to organogenesis: Application for phenotyping analysis of early embryonic lethality in mutant animals. Mamm. Genome.

[31] Hsu C.W., Kalaga S., Akoma U., Rasmussen T.L., Christiansen A.E., Dickinson M.E. (2019). High resolution imaging of mouse embryos and neonates with X-ray micro-computed tomography. Curr. Protoc. Mouse Biol..

[32] Busse M., Müller M., Kimm M.A., Ferstl S., Allner S., Achterhold K., Herzen J., Pfeiffer F. (2018). Three-dimensional virtual histology enabled through cytoplasm-specific X-ray stain for microscopic and nanoscopic computed tomography. Proc. Natl. Acad. Sci. USA..

[33] Kastner D.B., Kharazia V., Nevers R., Smyth C., Astudillo-Maya D.A., Williams G.M., Yang Z., Holobetz C.M., Santina L.D., Parkinson D.Y. (2020). Scalable method for micro-CT analysis enables large scale quantitative characterization of brain lesions and implants. Sci. Rep..

[34] Khimchenko A., Deyhle H., Schulz G., Schweighauser G., Hench J., Chicherova N., Bikis C., Hieber S.E., Müller B. (2016). Extending two-dimensional histology into the third dimension through conventional micro computed tomography. Neuroimage.

[35] Saccomano M., Albers J., Tromba G., Dobrivojević-Radmilović M., Gajović S., Alves F., Dullin C. (2018). Synchrotron inline phase contrast µCT enables detailed virtual histology of embedded soft-tissue samples with and without staining. J. Synchrotron. Rad..

[36] Chiani F., Orsini T., Gambadoro A., Pasquini M., Putti S., Cirilli M., Ermakova O., Tocchini-Valentini G.P. (2019). Functional loss of Ccdc1 51 leads to hydrocephalus in a mouse model of primary ciliary dyskinesia. Dis. Model. Mech..

[37] Massimi L., Pieroni N., Maugeri L., Fratini M., Brun F., Burkeeva I., Santamaria G., Medici V., Poloni T.E., Balducci C. (2020). Assessment of plaque morphology in Alzheimer’s mouse cerebellum using three-dimensional X-ray phase-contrast virtual histology. Sci Rep..

[38] Cedola A., Bravin A., Burkeeva I., Fratini M., Pacurenau A., Mittone A., Massimi L., Cloetens P., Coan P., Campi G. (2017). X-ray phase contrast tomography reveals early vascular alterations and neuronal loss in a multiple sclerosis model. Sci. Rep..

[39] Kuan A.T., Phelps J.S., Thomas L.A., Nguyen T.M., Han J., Chen C.L., Azevedo A.W., Tuthill J.C., Funke J., Cloetens P. (2020). Dense neuronal reconstruction through X-ray holographic nano-tomography. Nat. Neurosci..

[40] Schulz G., Weitkamp T., Zanette I., Pfeiffer F., Beckmann F., David C., Rutishauser S., Reznikova E., Müller B. (2010). High-resolution tomographic imaging of a human cerebellum: Comparison of absorption and grating-based phase contrast. J. R. Soc. Interface.

[41] Dyer E.L., Gray Roncal W., Prasad J.A., Fernandes H.L., Gürsoy D., De Andrade V., Fezzaa K., Xiao X., Vogelstein J.T., Jacobsen C. (2017). Quantifying mesoscale neuroanatomy using X-ray microtomography. eNeuro.

[42] Fonseca M.C., Araujo B.H.S., Dias C.S.B. (2018). High-resolution synchrotron-based X-ray microtomography as a tool to unveil the three-dimensional neuronal architecture of the brain. Sci. Rep..

[43] Müller M., Kimm M.A., Ferstl S., Allner S., Achterhold K., Herzen J., Pfeiffer F., Busse M. (2018). Nucleus-specific X-ray stain for 3D virtual histology. Sci. Rep..

[44] Dullin C., Ufartes R., Larsson E., Martin S., Lazzarini M., Tromba G., Missbach-Guentner J., Pinkert-Leetsch D., Katschinski D.M., Alves F. (2017). μCT of ex-vivo stained mouse hearts and embryos enables a precise match between 3D virtual histology, classical histology and immunochemistry. PLoS ONE.

[45] Töpperwien M., Markus A., Alves F., Salditt T. (2019). Contrast enhancement for visualizing neuronal cytoarchitecture by propagation-based x-ray phase-contrast tomography. Neuroimage.

[46] Shahmoradian S.H., Tsai E.H.R., Diaz A., Guizar-Sicarios M., Raabe J., Spycher L., Britschgi M., Ruf A., Stahlberg H., Holler M. (2017). Three-dimensional imaging of biological tissue by cryo X-ray ptychography. Sci. Rep..

[47] Bayguinov P.O., Fisher M.R., Fitzpatrick J.A. (2020). Assaying three-dimensional cellular architecture using X-ray tomographic and correlated imaging approaches. J. Biol. Chem..

[48] Metscher B.D., Müller G.B. (2011). MicroCT for molecular imaging: Quantitative visualization of complete three-dimensional distributions of gene products in embryonic limbs. Dev. Dyn..

[49] Hoshi M., Reginensi A., Joens M.S., Fitzpatrick J.A.J., McNeill H., Jain S. (2018). Reciprocal Spatiotemporally Controlled Apoptosis Regulates Wolffian Duct Cloaca Fusion. J. Am. Soc. Nephrol..

[50] Casadaban M.J., Cohen S.N. (1980). Analysis of gene control signals by DNA fusion and cloning in Escherichia coli. J. Mol. Biol..

[51] Chilvers K.F., Perry J.D., James A.L., Reed R.H. (2001). Synthesis and evaluation of novel fluorogenic substrates for the detection of bacterial beta-galactosidase. J. Appl. Microbiol..

[52] Tung C.H., Zeng Q., Shah K., Dong-Eog K., Schellingerhout D., Weissleder R. (2004). In vivo imaging of beta-galactosidase activity using far red fluorescent switch. Cancer Res..

[53] Louie A.Y., Huber M.M., Ahrens E.T., Rothbacher U., Moats R., Jacobs R.E., Fraser S.E., Meade T.J. (2000). In vivo visualization of gene expression using magnetic resonance imaging. Nat. Biotechnol..

[54] West D.B., Pasumarthi R.K., Baridon B., Djan E., Trainor A., Griffey S.M., Engelhard E.K., Rapp J., Li B., de Jong P.J. (2015). A lacZ reporter gene expression atlas for 313 adult KOMP mutant mouse lines. Genome Res..

[55] Graham J.M., Spencer A.H. (2010). Molecular and neuroimaging findings in pontocerebellar hypoplasia type 2 (PCH2): Is prenatal diagnosis possible?. Am. J. Med. Genet. A.

[56] Rudnik-Schöneborn S., Barth P.G., Zerres K. (2014). Pontocerebellar hypoplasia. Am. J. Med. Genet. C Semin. Med. Genet..

[57] Namavar Y., Barth P.G., Kasher P.R., van Ruissen F., Brockmann K., Bernert G., Writzl K., Ventura K., Cheng E.Y., Ferriero D.M. (2011). Clinical, neuroradiological and genetic findings in pontocerebellar hypoplasia. Brain.

[58] Namavar Y., Chitayat D., Barth P.G., van Ruissen F., de Wissel M.B., Poll-The B.T., Silver R., Baas F. (2011). TSEN54 mutations cause pontocerebellar hypoplasia type 5. Eur J. Hum. Genet..

[59] Cassandrini D., Biancheri R. (2010). Pontocerebellar hypoplasia: Clinical, pathologic, and genetic studies. Neurology.

[60] Van Dijk T., Baas F., Barth P.G., Poll-The B.T. (2018). What’s new in pontocerebellar hypoplasia? An update on genes and subtypes. Orphanet. J. Rare Dis..

[61] Paushkin S.V., Patel M., Furia B.S., Peltz S.W., Trotta C.R. (2004). Identification of a human endonuclease complex reveals a link between tRNA splicing and pre-mRNA 3′ end formation. Cell.

[62] Budde B.S., Namavar Y., Barth P.G., Poll-The B.T., Nurnberg G., Becker C., van Ruissen F., Weterman M.A., Fluiter K., te Beek E.T. (2008). tRNA splicing endonuclease mutations cause pontocerebellar hypoplasia. Nat. Genet..

[63] Breuss M.W., Sultan T., James K.N., Rosti R.O., Scott E., Musaev D., Furia B., Reis A., Sticht H., Al-Owain M. (2016). Autosomal-recessive mutations in the tRNA splicing endonuclease subunit TSEN15 cause pontocerebellar hypoplasia and progressive microcephaly. Am. J. Hum. Genet..

[64] Kasher P.R., Namavar Y., van Tijn P., Fluiter K., Sizarov A., Kamermans M., Grierson A.J., Zivkovic D., Baas F. (2011). Impairment of the tRNA-splicing endonuclease subunit 54 (tsen54) gene causes neurological abnormalities and larval death in zebrafish models of pontocerebellar hypoplasia. Hum. Mol. Genet..

[65] Pearson B., Wolf P.L., Vazquez J. (1963). A comparative study of a series of new indolyl compounds to localize beta-galactosidase in tissues. Lab. Invest..

[66] Cotson S., Holt S.J. (1958). Studies in enzyme cytochemistry. IV. Kinetics of aerial oxidation of indoxyl and some of its halogen derivatives. Proc. R. Soc. Lond. B Biol. Sci..

[67] Lojda Z., Kraml J. (1970). Indigogenic methods for glycosidases. I. An improved method for beta-D-glucosidase and its application to localization studies on intestinal and renal enzymes. Histochemie.

[68] Wiberg E., Wiberg N., Nils W.E. (2001). Inorganic Chemistry.

[69] Grosvenor A.P., Kobe B.A., McIntyre N.S. (2004). Examination of the oxidation of iron by oxygen using X-ray photoelectron spectroscopy and QUASES. Surface Science.

[70] Paxinos G., Franklin K., Paxinos G., Franklin K. (2004). The Mouse Brain in Stereotaxic Coordinates.

[71] Wang X., Zeng W., Yang X., Fang C., Han Y., Fei P. (2021). Bi-channel image registration and deep-learning segmentation (BIRDS) for efficient, versatile 3D mapping of mouse brain. Elife.

[72] Skarnes W.C., Rosen B., West A.P., Koutsourakis M., Bushell W., Iyer V., Mujica A.O., Thomas M., Harrow J., Cox T. (2011). A conditional knockout resource for the genome-wide stud of mouse gene function. Nature.

[73] Bradley A., Anastassiadis K., Ayadi A., Battey J.F., Bell C., Birling M.C., Bottomley J., Brown S.D., Bürger A., Bult C.J. (2012). The mammalian gene function resource: The international knockout mouse consortium. Mamm. Genome.

[74] Marcó S., Haurigot V., Bosch F. (2019). In vivo gene therapy for mucopolysaccharidosis Type III (Sanfilippo Syndrome): A new treatment horizon. Hum. Gene Ther..

[75] Meyer-Dilhet G., Courchet J. (2020). In utero cortical electroporation of plasmids in the mouse embryo. STAR Protoc..

[76] Epstein R.A. (2008). Parahippocampal and retrosplenial contributions to human spatial navigation. Trends Cogn Sci..

[77] Sugar J., Witter M.P., van Strien N.M., Cappaert N.L. (2011). The retrosplenial cortex: Intrinsic connectivity and connections with the (para)hippocampal region in the rat. Front. Neuroinform..

[78] Squire L.R., Stark C.E., Clark R.E. (2004). The medial temporal lobe. Annu. Rev. Neurosci..

[79] Dillingham C.M., Frizzati A., Nelson A.J., Vann S.D. (2015). How do mammillary body inputs contribute to anterior thalamic function?. Neurosci. Biobehav Rev..

[80] Kratochwil C.F., Maheshwari U., Rijli F.M. (2017). The long journey of pontine nuclei neurons: From rhombic lip to cortico-ponto-cerebellar circuitry. Front. Neur. Circuits.

[81] Di Pietro C., La Sala G., Matteoni R., Marazziti D., Tocchini-Valentini G.P. (2019). Genetic ablation of Gpr37l1 delays tumor occurrence in Ptch1+/− mouse models of medulloblastoma. Exp. Neurol..

