# Supplementary material for: Three-Dimensional X-ray Imaging of β-Galactosidase Reporter Activity by Micro-CT: Implication for Quantitative Analysis of Gene Expression
Source: Brain Sci. 2021 Jun 4;11(6):746. doi: 10.3390/brainsci11060746 (PMC8230009; doi:10.3390/brainsci11060746)

Video S1: Screenshot of the transfer function editor windows of the CTVOX analyser (Bruker software) demonstrates setting of the RGB transfer function curves for building a colour volume-rendered 3D model; colour coded for the tissue density function: red-blue-green; transparency level defined by the purple line.

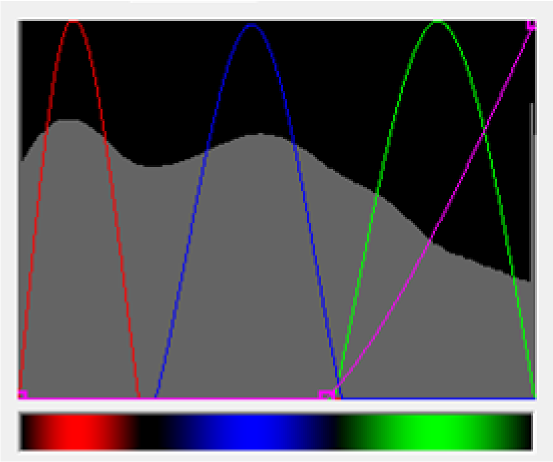

Video S2: Screenshot of the transfer function editor windows of the CTVOX analyser (Bruker software) demonstrates setting of the RGB transfer function curves for building a colour volume-rendered 3D model; colour coded for the tissue density function: red-blue-green; transparency level defined by the purple line.

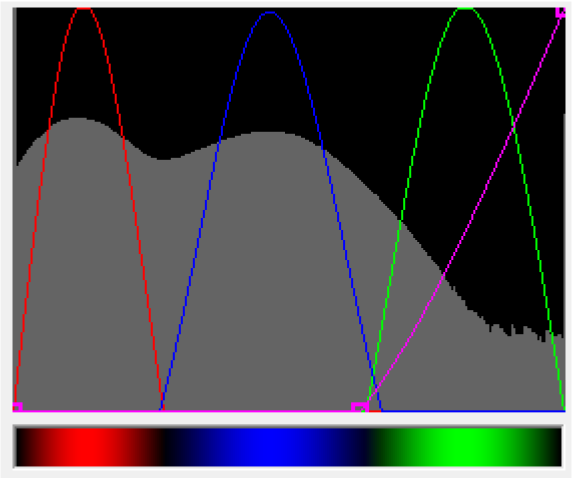

Supplement: Supplementary file 1 [file brainsci-11-00746-s001.zip › brainsci-1192088-supplementary/supplementary files/Supplementary info Movie S1 and Movie S2.pdf]
