# Supplementary figures and images for: Three-Dimensional X-ray Imaging of β-Galactosidase Reporter Activity by Micro-CT: Implication for Quantitative Analysis of Gene Expression
Source: Brain Sci. 2021 Jun 4;11(6):746. doi: 10.3390/brainsci11060746 (PMC8230009; doi:10.3390/brainsci11060746)

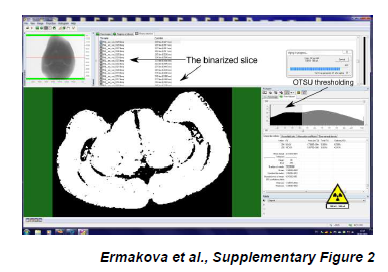

Supplement: Supplementary file 1 [file brainsci-11-00746-s001.zip › brainsci-1192088-supplementary/supplementary files/Suppl Fig 2.png]

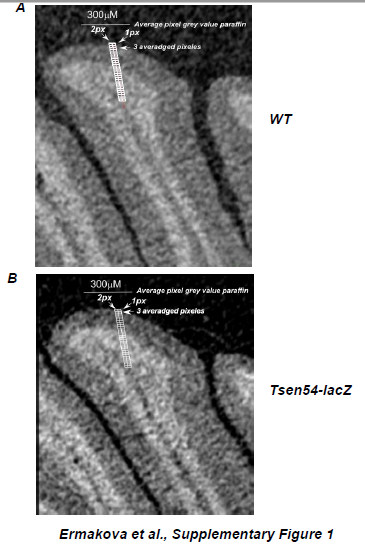

Supplement: Supplementary file 1 [file brainsci-11-00746-s001.zip › brainsci-1192088-supplementary/supplementary files/Suppl Figure 1.png]
